# Tonsillar carcinoma in dogs: Treatment outcome and potential prognostic factors in 123 cases

**DOI:** 10.1111/jvim.16623

**Published:** 2023-01-27

**Authors:** Elisabetta Treggiari, MacKenzie A. Pellin, Giorgio Romanelli, Gianluca Maresca, Irina Gramer, Andrew D. Yale, Evi Pecceu, Matteo Pignanelli, Juan Borrego, Katarzyna Purzycka, Davide Berlato

**Affiliations:** ^1^ Centro Specialistico Veterinario Milan Italy; ^2^ School of Veterinary Medicine University of Wisconsin Madison Wisconsin USA; ^3^ Queen Mother Hospital for Animals Royal Veterinary College Hertfordshire UK; ^4^ Davies Veterinary Specialists Higham Gobion Hitchin UK; ^5^ Royal Dick School, Swann Cancer Center University of Edinburgh Edinburgh UK; ^6^ Southfields Veterinary Specialists Essex UK; ^7^ Aùna Especialidades Veterinarias Paterna Valencia Spain; ^8^ Anderson and Moores Veterinary Specialists Winchester UK; ^9^ Dick White Specialists Referrals Six Mile Bottom Cambridgeshire UK; ^10^ Present address: Dierenartsencentrum Hond en Kat Deinze Belgium; ^11^ Present address: Lumbry Park Veterinary Specialists Hampshire UK; ^12^ Present address: Animal Oncology and Imaging Center Hunenberg CH Switzerland

**Keywords:** carcinoma, chemotherapy, dog, radiotherapy, surgery, tonsillar

## Abstract

**Background:**

Tonsillar carcinomas are rarely reported in dogs. Information on outcome after treatment is sparse and prognosis is guarded to poor.

**Hypothesis/Objectives:**

Assess treatment outcome and potential prognostic factors in a population of dogs with cytological or histopathological diagnosis of tonsillar carcinoma.

**Animals:**

A total of 123 client‐owned dogs with diagnosis of tonsillar carcinoma confirmed by cytology or histopathology.

**Methods:**

Retrospective, multi‐institutional study. Medical records of 12 institutions were reviewed from 2012 to 2021.

**Results:**

Treatment included surgery, chemotherapy (conventional, tyrosine kinase inhibitors or metronomic chemotherapy), radiotherapy, nonsteroidal anti‐inflammatory drugs (NSAIDs) or a combination of these. Surgery was performed in 68 cases, chemotherapy was administered in association with NSAIDs in 64 cases, NSAIDs were used alone in 14 cases and in association with surgery in 21 cases, whereas radiotherapy was used alone or in combination with surgery or chemotherapy in 20 cases. Overall survival time (OST) was 126 days (95% confidence interval [CI], 88‐164). Significantly longer survival (*P* < .001) was seen in dogs without evidence of metastatic disease (median survival time, 381 days; 95% CI, 116‐646). Other significant positive prognostic factors included absence of clinicals signs at presentation, surgery (tonsillectomy), use of adjuvant chemotherapy and use of NSAIDs.

**Conclusion and Clinical Importance:**

Asymptomatic dogs, those treated with surgery, those that received adjuvant chemotherapy, and those that received NSAIDs may have a better prognosis than previously expected, but overall survival remains short for dogs with tonsillar carcinoma.

**Abbreviations**
CA: carcinoma
CR: complete response
CT: computed tomography
GA: general anesthesia
GI: gastrointestinal
Gy: grays
KM: Kaplan‐Meier
MST: median survival time
MTD: maximum tolerated dose
NSAIDs: nonsteroidal anti‐inflammatory drugs
PD: progressive disease
PR: partial response
RT: radiotherapy
SCC: squamous cell carcinoma
SD: stable disease
TKIs: tyrosine kinase inhibitors
VCOG: Veterinary Cooperative Oncology Group

## 1 INTRODUCTION

Primary tonsillar carcinoma is reported rarely in dogs when compared to non‐tonsillar oral carcinomas and has a higher potential for local metastatic disease (up to 73%), when compared to oral carcinomas in other locations. Metastatic disease usually involves regional lymph nodes (mandibular, retropharyngeal, cervical) and lungs.^1^, ^2^ Lymph node metastasis has been reported in 9/11 dogs (82%) that had their lymph nodes examined, and in the same study 2/40 patients (5%) had suspected pulmonary metastasis.^1^ A more recent study found local lymph node metastasis in 7/15 dogs (47%), whereas 2/15 (13%) had nodal and distant (pulmonary) metastasis.^3^ The most common histological subtype is squamous cell carcinoma (SCC, 55%), followed by lymphoma (17%) and melanoma (12%).^4^, ^5^ Carcinoma (CA) also has been reported.^6^, ^7^

The etiology of this tumor is unclear, but it initially was suggested that dogs living in urban areas were prone to develop tonsillar neoplasia.^8^ A later epidemiologic study, however, did not find any difference in the incidence of tonsillar carcinoma in dogs living in smaller vs larger urban areas.^9^

Tumor diagnosis often is challenging because of the small size of the primary tumors, vague or no clinical signs, or incidental ventrolateral neck masses, consistent with an enlarged retropharyngeal lymph node, noted by the owners.^1^, ^3^ Oral cavity examination under general anesthesia (GA) or sedation, cytology or histopathology of the tonsil or affected lymph nodes and diagnostic imaging are helpful for tumor diagnosis. Staging procedures include contrast‐enhanced thoracic computed tomography (CT), thoracic radiographs and abdominal imaging (CT or ultrasound examination) with concurrent evaluation of local lymph nodes (mandibular, retropharyngeal, cervical).

Treatment options include surgery, radiotherapy (RT), chemotherapy or some combination of these, which resulted in a median survival time (MST) of 270 days in 22 dogs and 179 days in 44 dogs.^1^, ^6^ The most common chemotherapy drugs administered to dogs with tonsillar carcinomas are platinum agents or anthracyclines.^1^, ^3^, ^10^

Regardless of treatment, MST was short in early studies, ranging from 179 to 270 days.^1^, ^3^, ^10^ Previously identified negative prognostic factors include presence of clinical signs (e.g., lethargy, anorexia).^1^ Treatment with chemotherapy and RT was associated with longer MST, whereas clinical stage was not prognostic.^1^ Conversely, a more recent study found that dogs with unilateral involvement and no evidence of metastatic disease had a MST of 637 days, compared to 134 and 75 days for those patients with local or distant metastasis, respectively. The MST for patients treated using surgery and adjuvant chemotherapy was 464 days.^3^

Limited information is available with regard to patient outcome after various treatments, including surgery, chemotherapy, and radiation, for dogs with tonsillar carcinomas. Our aim was to report the outcome of dogs with tonsillar carcinoma that underwent different treatment modalities. A secondary aim was to assess prognostic factors influencing disease progression and overall survival.

## 2 MATERIALS AND METHODS

Ours was a retrospective, multi‐institutional study. Medical databases of 12 institutions were reviewed from 2012 to 2021 for dogs with a confirmed diagnosis of a primary tonsillar carcinoma either by cytology or histopathology. Information recorded included breed, sex, age, body weight, presenting clinical signs, site of tonsillar involvement, staging procedures, clinical stage, histological or cytological diagnosis and treatment (type of surgery, RT, chemotherapy and use of nonsteroidal anti‐inflammatory drugs [NSAIDs]).

Additional information included results of restaging procedures, including clinical and oral cavity examination, cytology, and diagnostic imaging when available. Restaging usually was performed every 3 to 6 months, depending on owner compliance and financial resources. Dogs were excluded if medical records were incomplete or if the diagnosis of primary tonsillar carcinoma was not confirmed by cytology or histopathology.

### 2.1 Treatment

Treatment included surgery, NSAIDs and other pain relief medications, chemotherapy, or RT, used alone or in combination.

Surgery consisted of tonsillectomy alone or in combination with lymphadenectomy. Dogs undergoing incisional biopsies were excluded from the group receiving surgery as a form of primary treatment. Radiotherapy and chemotherapy either were administered as primary treatment for those patients not undergoing surgical excision or as adjuvant treatment for microscopic disease after surgery.

Chemotherapy protocols could consist of induction or rescue treatment. Chemotherapy could include conventional, maximum tolerated dose (MTD) chemotherapy, tyrosine kinase inhibitors (TKIs), or metronomic chemotherapy, and it was used alone or in association with long‐term NSAIDs. Treatment included carboplatin (250‐300 mg/m^2^ IV every 3 weeks), doxorubicin (30 mg/m^2^ IV every 3 weeks), mitoxantrone (5 mg/m^2^ IV every 3 weeks) or cyclophosphamide‐based metronomic chemotherapy (10‐15 mg/m^2^ PO q 24 h). Tyrosine kinase inhibitors included toceranib phosphate (2.75‐3.25 mg/kg PO on a Monday/Wednesday/Friday schedule or every other day) or imatinib mesylate (8 mg/kg PO q 24 h). Maropitant was used (1 mg/kg IV or 2 mg/kg PO q 24 h) before or after conventional chemotherapy.

Radiotherapy was delivered as palliative or definitive treatment, alone or in combination with surgery, chemotherapy or NSAIDs. Palliative protocols consisted either of 32 Gy (8 Gy × 4) in 4 weekly fractions, 24 Gy (4 Gy × 6) in 6 daily fractions or 20 Gy (4 Gy × 5) in 5 daily fractions. Definitive‐intent treatments totaled 40 to 57 Gy delivered in 10 to 18 fractions.

Various NSAIDs (carprofen, piroxicam, meloxicam, firocoxib, and robenacoxib) were used at standard dosages, q24h, alone or in association with surgery, chemotherapy and RT. Palliative‐intent treatment included NSAIDs (q24h) or prednisolone (q24h), paracetamol or paracetamol and codeine (q12h) and tramadol (q12h or q8h), used PO alone at standard dosages, started at the time of diagnosis until disease progression or euthanasia.

### 2.2 Treatment response

Response to treatment was determined by the primary clinician's assessment, including clinical examination and oral cavity examination under GA or sedation, or using cytology in combination with diagnostic imaging (CT or thoracic radiographs to assess for local and distant metastasis). Response was evaluated according to the Veterinary Cooperative Oncology Group Response Evaluation Criteria in Solid Tumors (VCOG RECIST, version 1) and classified as complete response (CR), partial response (PR), progressive disease (PD) and stable disease (SD).^11^ Complete response was defined as the disappearance of all target lesions. Pathologic lymph nodes had to be <10 mm in the short axis. Partial response was defined as at least 30% reduction in the sum of diameters of target lesions, taking as reference the baseline sum. Progressive disease was defined as either the appearance of ≥1 new lesions or at least a 20% increase in the sum of diameters of target lesions, taking as reference the smallest sum on study. The sum also must have shown an absolute increase of 5 mm. Stable disease was defined as <30% reduction (PR) or 20% increase (PD) in the sum of diameters of target lesions, taking as reference the smallest sum of diameters while on study.

Chemotherapy toxicity was graded according to the VCOG—Common Terminology Criteria for Adverse Events (VCOG‐CTCAE, version 2).^12^ Dose reductions when toxicity occurred were performed at the clinician's discretion. Classification of adverse events (AEs) was done retrospectively by reviewing medical records, as assessed by the clinician in charge of the case at the time of each visit.

### 2.3 Clinical signs

Clinical signs at presentation were categorized as respiratory (e.g., cough, dyspnea, upper respiratory noises, stertorous breathing, stridor, reverse sneezing), gastrointestinal (GI; e.g., decreased appetite or anorexia, vomiting, diarrhea, ptyalism, dysphagia) or a combination of respiratory and GI. Additional constitutional (e.g., lethargy, weight loss) and neurological (e.g., neck pain, head tilt, facial nerve paralysis, behavioral changes, anisocoria, dysphonia) signs were documented if present. If the tumor was an incidental finding at presentation, clinical signs were not classified. If signs were not categorized as respiratory, GI, neurological or constitutional, they were classified as other, including halitosis, retching, oral pain, oral discharge, lymphadenomegaly, neck edema, presence of a mandibular mass, or pyrexia.

### 2.4 Statistical analysis

Clinical data and follow‐up information were acquired from medical records or phone conversations with the referring veterinarians. If patients were lost to follow‐up, they were right censored at the time of their last visit or update.

The following characteristics were analyzed for statistical purposes and as potential prognostic factors: institution, breed, sex, age (categorized in quartiles), weight (categorized in quartiles), clinical signs at presentation (present or absent), tonsillar involvement (left or right; unilateral or bilateral), clinical stage (no metastasis detected; single metastatic lymph node; multiple metastatic lymph nodes or distant metastatic disease), surgery type (tonsillectomy with or without lymphadenectomy); incisional biopsy only or no surgery, RT (palliative or definitive intent; not performed), chemotherapy type (MTD; TKIs or metronomic chemotherapy; no chemotherapy), chemotherapy timing (as sole treatment; as an adjuvant to surgery or radiotherapy; no chemotherapy), and use of NSAIDs (prescribed; not prescribed). Prognostic factors were evaluated using the Kaplan‐Meier (KM) method and the associated log‐rank test.

The MST was defined as the time from diagnosis until death because of any cause, whereas progression‐free survival (PFS) was defined as the time from diagnosis until disease progression developed. A KM product‐limit method was used to estimate survival and the log‐rank test was applied to test differences between groups. All reported *P*‐values were 2‐sided, and a 5% significance level was set (*P* < .05). Multivariate Cox's proportional hazards regression analysis using a forward stepwise procedure was performed for factors significant on univariate analysis. Statistical analysis was performed using commercial software (IBM SPSS Statistics, version 23.0).

## 3 RESULTS

### 3.1 Study population

A total of 123 dogs were included in the study. Patients were classified as neutered males (n = 58, 49%), intact males (n = 27, 20%), neutered females (n = 31, 25%) and intact females (n = 7, 6%). Median body weight was 17.8 kg (range, 2‐48 kg) and median age was 10 years (range, 4‐15 years).

Patient demographics are summarized in Table 1.

**TABLE 1:** Patient demographics of 123 dogs with tonsillar carcinoma and summary of treatment(s) administered

Breed	Results
Purebred n = 96 (78%) Cross breed n = 27 (22%)	

Purebred	Number (%)
Cavalier King Charles spaniel	n = 17 (14)
English springer spaniel	n = 15 (12)
Border collie	n = 12 (10)
Bichon frise	n = 8 (7)
West Highland white terrier	n = 5 (4)
Other terrier breeds	n = 7 (6)
Chihuahua	n = 3 (2)
Weimaraner	n = 3 (2)
Retrievers	n = 3 (2)
Beagle	n = 2 (2)
Hungarian Vizla	n = 2 (2)
French bulldog	n = 2 (2)
English cocker spaniel	n = 2 (2)
German Shepherd	n = 2 (2)
Boxer	n = 1 (1)
Miniature Schnauzer	n = 1 (1)
Czechoslovakian wolf	n = 1 (1)
Poodle	n = 1 (1)
Dachshund	n = 1 (1)
Rough collie	n = 1 (1)
Bearded collie	n = 1 (1)
English setter	n = 1 (1)
Grand basset griffon Vendeen	n = 1 (1)
Nova Scotia Duck‐tolling retriever	n = 1 (1)
Portuguese Water Dog	n = 1 (1)
Podenco canario	n = 1 (1)
Catalan sheepdog	n = 1 (1)

### 3.2 Clinical signs, diagnosis, and staging

Presenting clinical signs were GI (n = 34, 28%), respiratory (n = 17, 14%), respiratory and GI (n = 20, 16%), and other (n = 49, 40%). Twenty patients (16%) additionally had constitutional and 13 (11%) neurological signs. In 3 cases (2%), the tumor was an incidental finding at the time of GA and intubation before a scheduled surgical procedure. One‐hundred and fifteen dogs (94%) had laboratory blood tests performed: hematology and biochemistry were performed in 109 cases, whereas 2 cases had hematology only and 4 biochemistry only; urinalysis was performed in 12 cases and no abnormalities were found. Hematology and biochemistry abnormalities are summarized in Table 2.

**TABLE 2:** Hematology and biochemistry abnormalities identified in 81/115 dogs with tonsillar carcinoma

Test	No. of patients with abnormal results (%)	Abnormalities: 81/115 dogs					
Hematology	37 (32%)	Leukocytosis (10) Neutrophilia (11) Neutropenia (2)	Lymphocytosis (4) Lymphopenia (6)	Monocytosis (5) Monocytopenia (1)	Low HCT (7) Increased HCT (1)	Thrombocytosis (7) Thrombocytopenia (3)	Eosinophilia (1)
Biochemistry	45 (39%)	ALT elevation (11) ALP elevation (21) AST elevation (3) Increased GGT (2)	Increased total bilirubin (1) Increased bile acids (2)	Elevation of creatinine kinase (1) Elevation blood urea nitrogen (2) Decrease blood urea nitrogen (2)	Decreased albumin (5) Hyperglobulinemia (9) Hyperproteinemia (1)	Increased cholesterol (2) Decreased cholesterol (1) Increased triglycerides (2) Elevation in LDH (1)	Hypokalemia (5) Hypochloremia (1) Hyperglycemia (2)

The tonsillar carcinoma was localized in the left tonsil in 61 cases (50%), in the right tonsil in 54 cases (44%), unilateral in 115 cases and bilateral in 7 cases (6%). The location was not reported in 1 case (1%). The primary diagnosis was achieved by cytology in 28 cases (23%), histopathology in 82 (67%) and both in 13 cases (11%). Diagnosis was consistent with SCC in 102 cases (83%) and with carcinoma in 21 dogs (17%). Histopathological diagnosis was achieved after tonsillectomy in 67 cases (54%) and by incisional biopsy in 15 cases (12%).

Staging included contrast‐enhanced CT of head, neck and thorax in 94 dogs (76%). Thoracic radiographs were performed in conjunction with thoracic CT in 6 cases (5%). Thoracic radiography as the only staging modality to assess for pulmonary metastases was performed in 26 cases (21%). Abdominal imaging was performed in 31/123 cases (25%). Abdominal CT was performed in 23 cases (74%), abdominal ultrasound examination in 6 cases (19%) and abdominal radiographs in 2 cases (6%).

The remainder of patients were staged using a combination of head CT (n = 5), head magnetic resonance imaging (MRI, n = 2), or thoracic CT and neck ultrasound examination (n = 1). One patient did not undergo any staging procedures.

Regional lymph nodes (mandibular, superficial cervical, and retropharyngeal) were examined by means of cytology or histopathology in 94 dogs (76%), if they were found to be enlarged on physical examination or in case of abnormal findings on CT, and not sampled in 29 dogs (19%). Of those sampled, 77 (82%) had regional metastasis, 16 (13%) were negative for metastatic disease and, in 1 case, cytology was not diagnostic. In 40 dogs (33%), only 1 lymph node was involved; multiple lymph nodes were affected by metastases in 35 dogs (29%). Nineteen dogs (15%) had distant (pulmonary, n = 19 and splenic, n = 1) metastatic disease, presumed or confirmed by cytology. Overall, staging procedures were negative for local or distant metastatic disease in 29 dogs (24%). Abdominal imaging did not identify any abnormalities consistent with distant metastases in 30 cases, whereas in 1 case splenic metastases were identified.

Restaging and assessment of clinical response were based on physical examination and oral cavity examination under GA or sedation (n = 33, 27%), CT scan of head, neck and thorax (n = 12, 10%), lymph node cytology (n = 4, 3%), thoracic radiographs (n = 4, 3%) and neck ultrasound examination (n = 1, 1%).

### 3.3 Treatment and response

Treatments administered are summarized in Table 3. Surgery was used in 68 dogs (55%) and consisted of tonsillectomy only in 30 dogs (44%), of which 23 (77%) underwent unilateral and 7 (23%) bilateral tonsillectomy, tonsillectomy and lymphadenectomy in 37 dogs (30%), of which 21 (57%) underwent unilateral and 16 (43%) bilateral tonsillectomy, and lymphadenectomy only in 1 dog (1%). Incisional biopsy was performed in 15 dogs (12%) and 40 dogs (32%) had no surgical procedure performed. Six dogs (9%) received surgery alone and 21 (31%) were treated using surgery and NSAIDs alone. Adjuvant, MTD chemotherapy (carboplatin) was used in 28 cases (41%), TKIs in 10 cases (14%), metronomic chemotherapy in 2 cases (3%), whereas adjuvant radiotherapy was used in 8 dogs (12%, of which 7 also were treated using chemotherapy).

**TABLE 3:** Summary of treatments administered to 123 dogs with tonsillar carcinoma

Treatment(s) administered	Number of patients
Surgery alone	6
Surgery and NSAIDs alone	21
Surgery and adjuvant antineoplastic agents	40
Surgery and adjuvant radiotherapy	1
Surgery, radiotherapy and antineoplastic agents	7
MTD chemotherapy alone	6
Toceranib alone	18
Antineoplastic agents in combination with NSAIDs	64
Radiotherapy alone	2
Radiotherapy and antineoplastic agents	10
Radiotherapy and NSAIDs	3
NSAIDs alone	14

Abbreviations: MTD, maximum tolerated dose; NSAIDs, nonsteroidal anti‐inflammatory drugs.

Radiotherapy was performed in 20 dogs (2%). For dogs treated with radiotherapy, 17 (85%) received a palliative protocol and 3 (15%) received a definitive protocol. Radiotherapy alone was used palliatively in 2 dogs (10%) and as an adjuvant to surgery in 1 case. Carboplatin and radiotherapy were used concurrently in 6 dogs (30%) of which 5 (83%) were treated palliatively and 1 (17%) received definitive treatment. Tyrosine kinase inhibitors were used concurrently in 4 dogs (20%) receiving a palliative protocol. Seven dogs (35%) received multimodal treatment including surgery (n = 4, tonsillectomy; n = 3, tonsillectomy and lymphadenectomy), radiotherapy (n = 5, palliative treatment; n = 2, definitive treatment) and chemotherapy (n = 4, carboplatin; n = 1, toceranib phosphate; n = 1, carboplatin and then toceranib phosphate; n = 1, metronomic cyclophosphamide). Overall responses, when determined, included CR (n = 2, 10%), PR (n = 5, 25%), SD (n = 1, 5%) and PD (n = 2, 10%).

Systemic anticancer treatment was administered to 76 dogs (62%): 42 patients (55%) received MTD chemotherapy (n = 40, carboplatin, n = 1, doxorubicin, n = 1, mitoxantrone), 32 (44%) TKIs (n = 30, toceranib phosphate, n = 2, imatinib mesylate) and 2 (3%) were treated with cyclophosphamide‐based metronomic chemotherapy. Conventional MTD chemotherapy was used as sole treatment in 6 dogs (8%) and included doxorubicin and mitoxantrone in 1 case each and carboplatin in 4 cases. Toceranib phosphate was used alone in 18 cases (24%) and imatinib mesylate in 1 case (1%).

Response to treatment with carboplatin included SD (n = 8 cases, 21%), PR (n = 3 cases, 8%) and PD (n = 12 cases, 31%). In the remainder of cases, a clinical response could not be determined because of lack of follow‐up. Response to toceranib phosphate as a primary treatment included SD (n = 7 cases, 21%), PR (n = 3 cases, 9%) and PD (n = 9 cases, 27%). Toxicity based on VCOG scale could be assessed for 75 patients and is summarized in Table 4.

**TABLE 4:** Adverse events identified in 75/123 dogs with tonsillar carcinoma receiving treatment with antineoplastic agents

Drug administered	No. of events	Type of toxicity	Description of toxicity	VCOG^a^ toxicity grade (G)
Carboplatin	15	Hematological	Neutropenia	G1 (n = 3); G2 (n = 2); G3 (n = 1); G4 (n = 3)
			Thrombocytopenia	G1 (n = 3); G2 (n = 2); G3 (n = 1)
	9	Gastrointestinal	Anorexia, nausea, diarrhea, vomiting, inappetance	G1 (n = 4); G2 (n = 4); G3 (n = 1)
	2	Miscellaneous	Dermatitis	G3
			Lethargy	G1
Mitoxantrone	1	Gastrointestinal	Anorexia	G1
Doxorubicin	1	Hematological	Neutropenia	G3
	1	Gastrointestinal	Nausea, inappetance	G1
Metronomic cyclophosphamide	1	Miscellaneous	Sterile hemorrhagic cystitis	G3
Toceranib phosphate	10	Gastrointestinal	Inappetence, nausea, diarrhea	G1 (n = 7); G2 (n = 2); G3 (n = 2)
	1	Miscellaneous	Lethargy	G3
			Creatinine elevation	G1
Imatinib mesylate	1	Gastrointestinal	Diarrhea	G1

^a^ Toxicity was categorized according to the Veterinary Co‐operative Oncology Group (VCOG) scheme.

Nonsteroidal anti‐inflammatory drugs were administered to 102 dogs (83%), and 2 patients (2%) were changed to a different NSAID during their treatment course. Patients were treated with meloxicam (n = 60, 59%), piroxicam (n = 17,17%), carprofen (n = 15, 15%), firocoxib (n = 7, 7%) robenacoxib (n = 3, 3%) and deracoxib (n = 1, 1%). The NSAIDs were used alone (n = 14, 14%) or in combination with chemotherapy (n = 64, 58%), for a median of 151 days (range, 5‐953 days). Specifically, NSAIDs were used in association with MTD chemotherapy in 35 cases (55%), with TKIs in 27 cases (42%) and with metronomic chemotherapy in 2 cases (3%). They were used with RT in 3 cases (3%). Adverse events included grade 1 vomiting and diarrhea in 2 cases (2%).

Dogs received prednisolone alone (n = 8, 7%), tramadol (n = 10, 1%), paracetamol and paracetamol/codeine alone (n = 7 cases, 6%) or in combination with NSAIDs (n = 3 cases, 2%), and gabapentin (n = 5, 4%), combined with other treatments. The remainder of patients did not receive any anti‐inflammatory or analgesic treatment.

Rescue treatment was used in 11 cases, after recurrence or metastatic disease was detected at the time of restaging, and consisted of cisplatin (n = 1), doxorubicin (n = 1), cyclophosphamide‐based metronomic chemotherapy (n = 3), toceranib phosphate (n = 4) and imatinib mesylate (n = 2). All cases developed PD based on physical examination or imaging findings during a time interval of 15 to 440 days.

### 3.4 Survival analysis and prognostic factors

**FIGURE 1:**
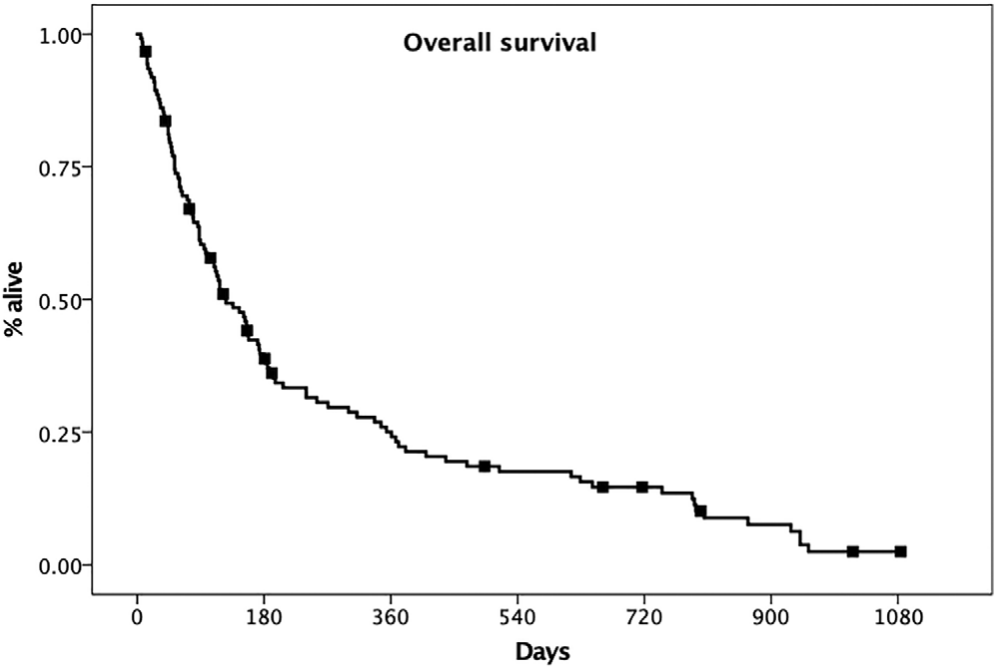
Kaplan‐Meier plot depicting the overall survival time calculated for 123 dogs with tonsillar carcinoma (126 days, 95% CI 88‐164). Censored patients are identified by a black mark

One‐hundred and nine dogs (89%) died, and 14 dogs (11%) were still alive or lost to follow‐up at the time of writing and therefore were censored. The median follow‐up time for censored dogs was 186 days (range, 12‐1084 days). All dogs died or were euthanized because of PD and the cause of death was tumor‐related in all cases. The overall survival time was 126 days (95% CI, 88‐164, Figure 1).

Prognostic factors and their association with survival time are summarized in Table 5.

**TABLE 5:** Variables analyzed for correlation with prognosis in 123 dogs with tonsillar carcinoma

Variable	Categories	No. of dogs	MST [95% CI]	*P* *
Clinical signs	Present	92	113 [78‐148]	.04
	Absent	31	185 [141‐230]	
Clinical stage	No metastatic disease	29	381 [116‐646]	<.001
	One metastatic lymph node	40	153 [64‐242]	
	Multiple metastatic lymph nodes or distant metastasis	54	88 [40‐136]	
Surgery	Tonsillectomy +/− lymphadenectomy	68	196 [106‐286]	.001
	Incisional biopsy or no surgery	55	88 [67‐109]	
Chemotherapy	Sole treatment	25	109 [71‐147]	.001
	Adjuvant chemotherapy	50	207 [53‐361]	
	No chemotherapy	48	109 [0‐240]	
Use of nonsteroidal anti‐inflammatory drugs	Yes	102	151 [105‐197]	.03
	No	21	54 [27‐81]	

* Significance was set at <.05.

**FIGURE 2:**
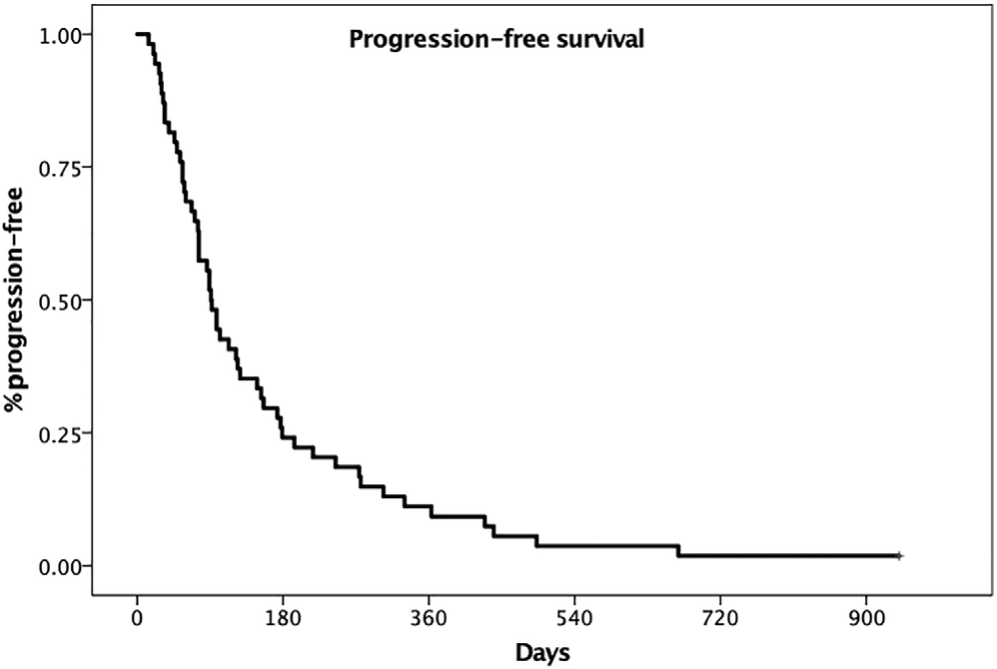
Kaplan‐Meier plot depicting the median progression free‐survival for dogs with tonsillar carcinoma (91 days, 95% CI 87‐105)

The median PFS (Figure 2) was calculated for 54 dogs and was 91 days (95% CI, 87‐105). There was no statistically significant association with the prognostic factors evaluated for overall survival.

**FIGURE 3:**
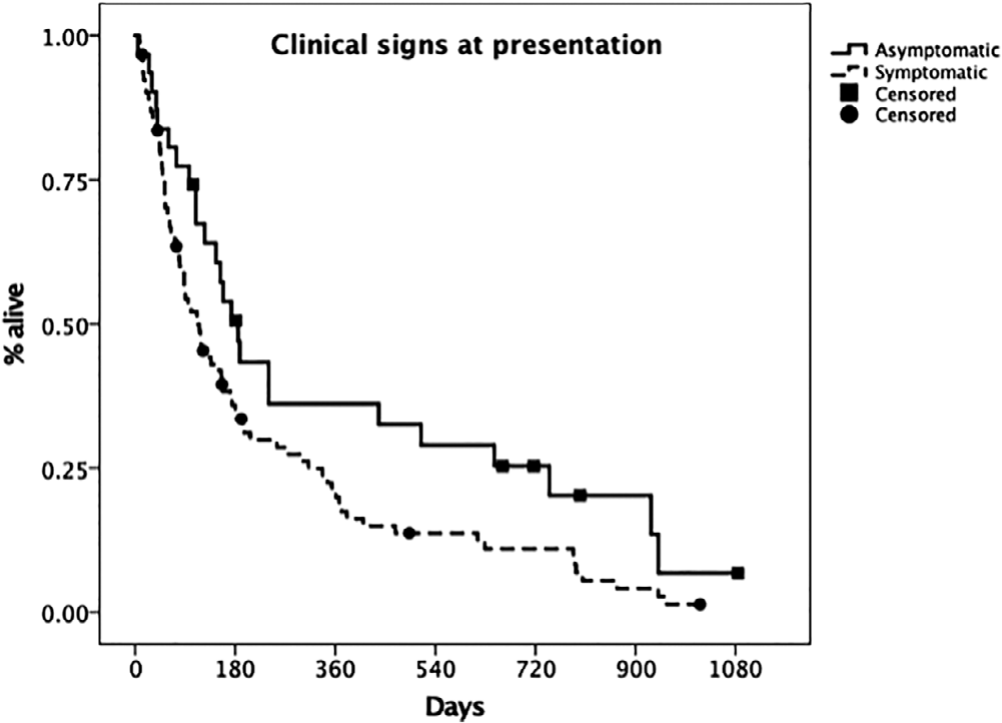
Kaplan‐Meier plot depicting the median survival time (MST) for asymptomatic (solid line) vs symptomatic patients (dashed line). The MST was 185 days (95% CI 141‐230) vs 113 days (95% CI 78‐148), respectively. The difference between groups was statistically significant (*P* = .04). Censored patients are identified by a black mark

Institution (*P* = .69), signalment (breed, *P* = .41; sex, *P* = .90; age, *P* = .67), and body weight (*P* = .28) did not significantly affect survival. Dogs with clinical signs at presentation had a significantly shorter MST than dogs without clinical signs (113 days, 95% CI, 78‐148 vs 185 days, 95% CI, 141‐230, respectively, *P* = .04, Figure 3). Site of tonsillar involvement (*P* = .13) and tumor type on cytology or histopathology (*P* = .09) were not significant.

**FIGURE 4:**
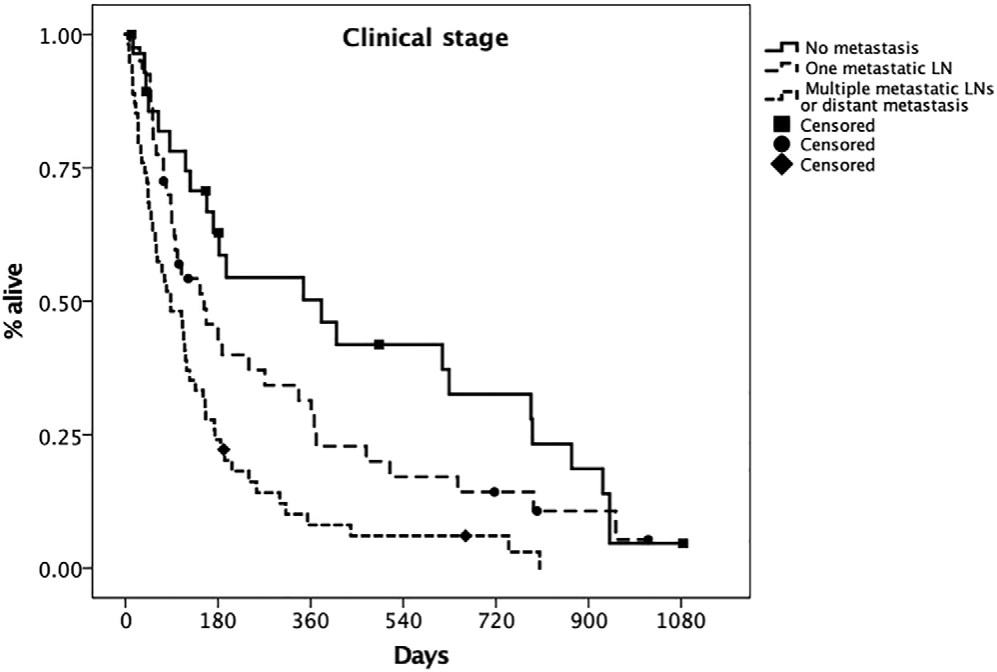
Kaplan‐Meier plot depicting the median survival time (MST) according to clinical stage. The MST in dogs without evidence of metastatic disease (solid line) was 381 days (95% CI 116‐646); in dogs with involvement of a single lymph node (dashed line) the MST was 153 days (95% CI 64‐242) and in dogs with involvement of multiple lymph nodes or distant metastatic disease (dotted line) the MST was 88 days (95% CI 40‐136). The difference between groups was statistically significant (*P* < .001). Censored patients are identified by a black mark

Clinical stage at the time of presentation was prognostic (Figure 4; *P* < .001). Significantly longer survival was seen in dogs without evidence of metastatic disease (MST, 381 days; 95% CI, 116‐646) compared to dogs with involvement of a single lymph node (MST, 153 days; 95% CI, 64‐242) and dogs with involvement of multiple lymph nodes or distant metastatic disease (MST, 88 days; 95% CI, 40‐136).

**FIGURE 5:**
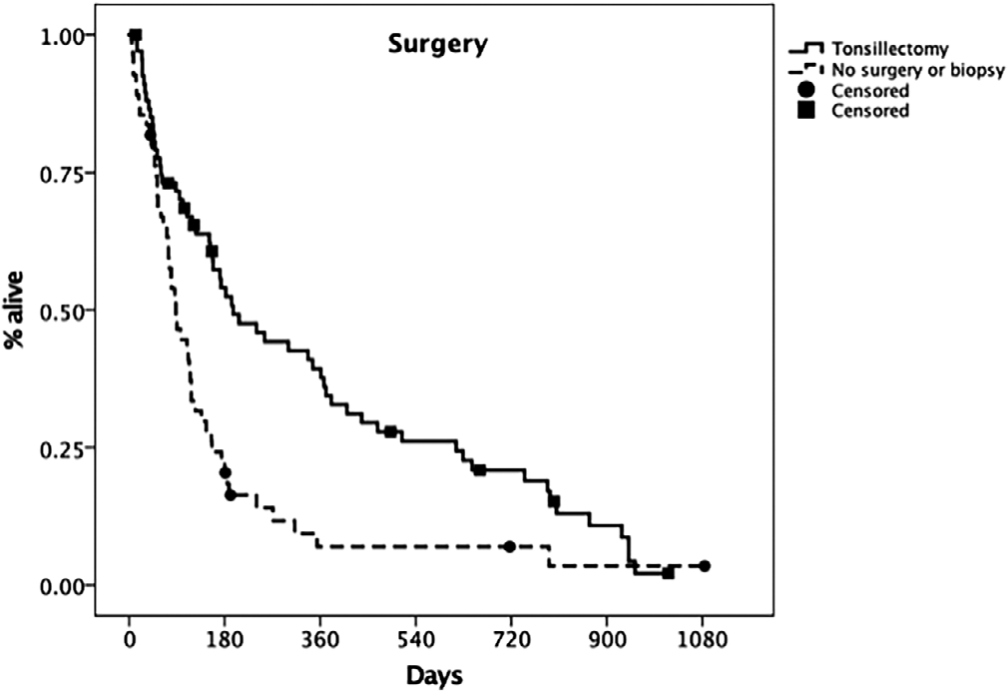
Kaplan‐Meier plot depicting the median survival time (MST) for dogs treated with tonsillectomy (solid line) vs dogs undergoing no surgery or incisional biopsy (dashed line). The MST was 196 days (95% CI 106‐286 days), vs 88 days (95% CI 67‐109 days), respectively. The difference between groups was statistically significant (*P* = .001). Censored patients are identified by a black mark

Regarding treatment, tonsillectomy with or without regional lymphadenectomy was related to a significantly longer survival (MST, 196 days; 95% CI, 106‐286 days) compared to incisional biopsy or no surgery (MST, 88 days; 95% CI, 67‐109 days). The difference was statistically significant (Figure 5, *P* = .001). Treatment with RT, regardless of the protocol used, did not influence survival (*P* = .44).

**FIGURE 6:**
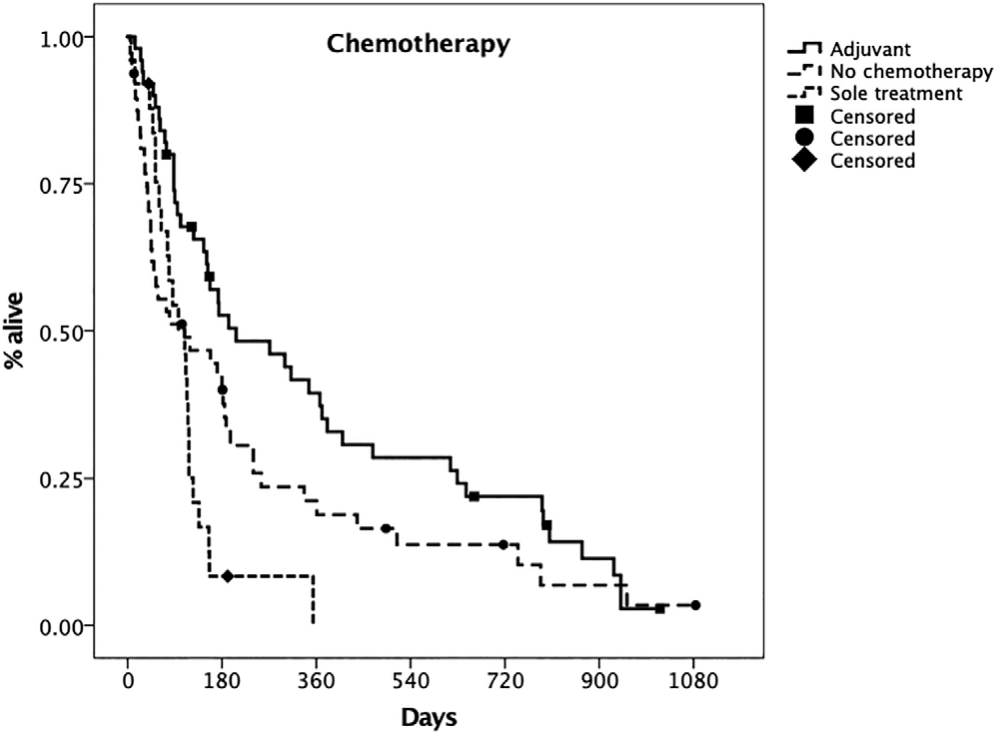
Kaplan‐Meier plot depicting the median survival time (MST) for dogs treated with adjuvant chemotherapy (solid line) vs. dogs undergoing no chemotherapy (dashed line) or chemotherapy alone (dotted line). The MST was 207 days (95% CI 53‐361) vs. 109 days (95% CI 0‐240 days) vs. 109 days (95% CI 71‐147), respectively. The difference between groups was statistically significant (*p* = 0.001). Censored patients are identified by a black mark

Use of chemotherapy (MTD vs metronomic chemotherapy or TKIs vs no chemotherapy) did not show any significant difference between groups (*P* = .17), but when chemotherapy timing was analyzed (adjuvant vs sole treatment vs no chemotherapy), the difference was significant (MST, 207 days; 95% CI, 53‐361 vs 109 days; 95% CI, 71‐147 days vs 109 days; 95% CI, 0‐240, respectively, *P* = .001, Figure 6).

**FIGURE 7:**
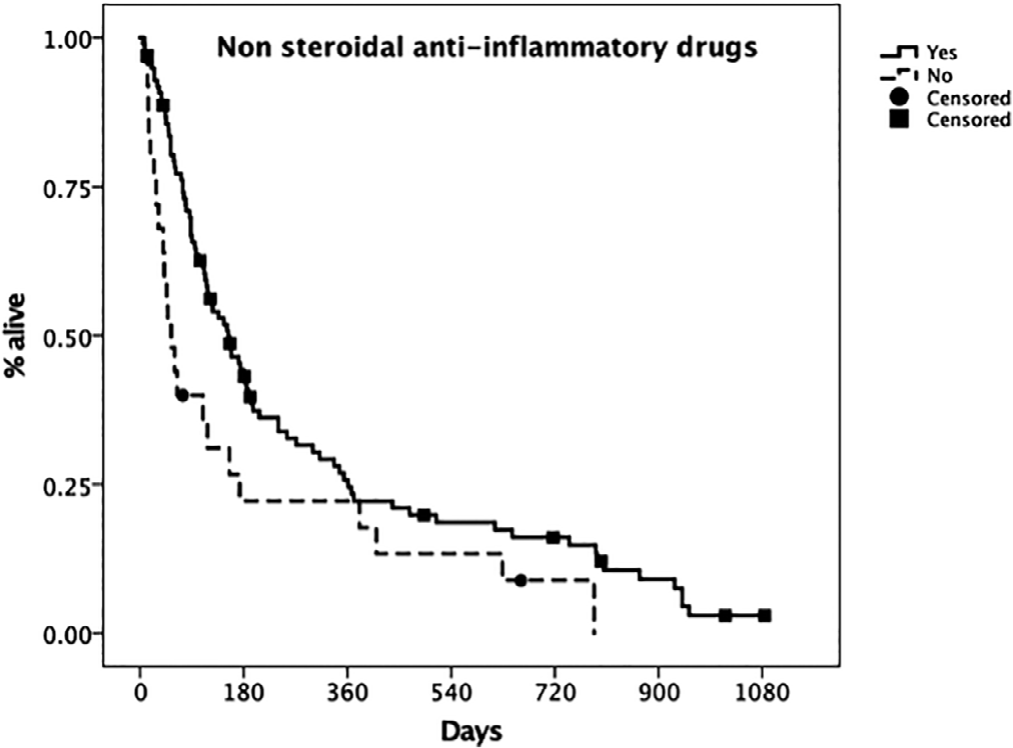
Kaplan‐Meier plot depicting the median survival time (MST) for dogs treated with nonsteroidal anti‐inflammatory drugs (solid line) vs untreated dogs (dashed line). The MST was 151 days (95% CI 105‐197) vs 54 days, (95% CI 27‐81), respectively. The difference between groups was statistically significant (*P* = .03). Censored patients are identified by a black mark

Use of NSAIDs also significantly affected MST (151 days; 95% CI, 105‐197) compared to dogs not receiving NSAIDs (54 days; 95% CI, 27‐81; *P* = .03; Figure 7).

Statistically significant factors for the univariate model, all of which were included in a multivariate model, independently influenced survival (Table 6). Negative prognostic factors were the presence of clinical signs at presentation (hazard ratio [HR], 2.1; 95% CI, 1.3‐3.3; *P* = .003) or metastatic disease, specifically 1 lymph node (HR, 1.7; 95% CI, 1.0‐3.0), and multiple lymph nodes or distant metastasis (HR, 2.7; 95% CI, 1.6‐4.7), *P* = .002. Positive prognostic factors were tonsillectomy with or without lymphadenectomy (HR, 0.5; 95% CI, 0.3‐0.9; *P* = .01) and use of NSAIDs (HR, 0.5; 95% CI, 0.3‐0.8; *P* = .004).

**TABLE 6:** Forward stepwise multivariate Cox's proportional hazards regression analysis of significant prognostic factors in 123 dogs with tonsillar carcinoma

Variable	Categories	HR [95% CI]	*P* *
Clinical signs	Absent	Ref	.003
	Present	2.1 [1.3‐3.3]	
Clinical stage	No metastatic disease	Ref	.002
	One metastatic lymph node	1.7 [1.0‐3.0]	
	Multiple metastatic lymph nodes or distant metastasis	2.7 [1.6‐4.7]	
Surgery	Incisional biopsy or no surgery	Ref	.01
	Tonsillectomy +/− lymphadenectomy	0.5 [0.3‐0.9]	
Chemotherapy	Adjuvant	Ref	.08
	Sole treatment	1.4 [0.8‐2.7]	
	No chemotherapy	0.9 [0.5‐1.7]	
Nonsteroidal anti‐inflammatory drugs	No	Ref	<.004
	Yes	0.5 [0.3‐0.8]	

* Significance was set at <.05.

The only factor not retaining significance in the multivariate analysis was chemotherapy (adjuvant vs sole treatment vs no chemotherapy; *P* = .08).

## 4 DISCUSSION

Our aim was to describe the outcome of dogs with tonsillar carcinoma treated with surgery, RT, chemotherapy or NSAIDs, used alone or in combination. In some cases, anti‐inflammatory drugs or pain relief medications were the only treatment prescribed. Results showed that absence of clinical signs at presentation, use of surgery and adjuvant chemotherapy and use of NSAIDs were related to significantly longer survival.

Our study had several limitations inherent to retrospective studies, including heterogeneity of staging procedures, clinician bias when recommending treatment, absence of follow‐up in some cases and use of anti‐inflammatory medications alone with more aggressive and invasive tumors. Lack of advanced imaging in some cases may have influenced staging because metastatic disease may have been overlooked.

Our study population included 123 dogs. Interestingly, some breeds seemed overrepresented, particularly spaniels and collies. This observation is consistent with previous studies, and these breeds could be predisposed to developing tonsillar cancer.^1^, ^3^ Male dogs also were overrepresented, as previously reported.^1^

Presence of clinical signs at the time of presentation significantly affected survival. This observation positively correlates with the findings of a previous study where lethargy and anorexia were related to a shorter survival time (103 and 22 days, respectively) and may reflect more aggressive and invasive tumors.^1^

Dogs were staged using a variety of different procedures, consistent with the retrospective and multicenter nature of our study, the time frame, and the owners' financial resources. The value of CT in assessing solid tumors and suspected metastasis has been reported for oral cancer, particularly with respect to regional lymph nodes, and the tomographic appearance of tonsillar tumors recently has been described.^5^, ^13^ In some cases, lack of advanced imaging could have influenced the determination of clinical stage, considering that 26 dogs were assessed for the presence of pulmonary metastasis using thoracic radiographs alone and, in 90 cases, abdominal imaging was not performed. Therefore, metastatic disease may have been underestimated.

Metastatic disease was common, with 82% of dogs presenting with regional metastasis and 15% with distant metastasis. This observation confirms the findings of previous studies where local metastatic disease was present in a higher proportion of dogs when compared to non‐tonsillar oral epithelial cancer.^6^ Abdominal imaging was performed in 31 cases and showed evidence of distant metastatic disease to the spleen in a single case. The tonsils recently have been reported to be a metastatic site from tumors not only located in the head and neck, but also elsewhere in the body.^4^ However, primary tumors and specifically carcinomas, were located in the head and neck in 54% of cases, whereas primary, metastatic tumors were located in the abdominal cavity in 1/53 cases (2%) and compatible with hemangiosarcoma of the liver.^4^ Given that our cases all were diagnosed as carcinoma, it is unlikely that we would have missed a primary epithelial cancer located in the abdominal cavity. However, it is recommended to always include abdominal imaging as a staging procedure to rule out other primary tumors and concurrent conditions. Therefore, metastatic disease may have been underestimated.

Metastasis did seem to affect survival, particularly in cases of regional metastatic disease. This finding could be related to the severity of clinical signs associated with lymphadenomegaly and its effects on quality of life when compared to asymptomatic or mildly symptomatic dogs. Additionally, in some cases, local metastatic disease could be associated with more invasive inoperable tumors, rapid disease progression, owners declining surgical treatment or the decision to euthanize. Histopathological examination of bilateral mandibular and medial retropharyngeal lymph nodes previously has been recommended to confirm metastatic disease, but not all of our cases underwent bilateral lymphadenectomy or had cytology of the contralateral lymph node performed, especially if lymph nodes were normal on CT.^14^ Sentinel lymph node mapping also has been shown to be helpful in identifying metastatic lymph nodes in dogs with oral tumors, and could similarly be considered when staging patients with tonsillar cancer.^4^, ^15^, ^16^

The same applies to the contralateral tonsil, which was not evaluated in all cases. It is known that the contralateral tonsil can be affected, and a previous study found that 3/5 dogs that had their contralateral tonsil examined had bilateral disease.^1^ Based on this observation, it is likely that the percentage of dogs with bilateral disease was underestimated in our study.

Dogs without local or distant metastases in our study had an MST of 381 days. These patients likely had a significantly longer MST because of slowly progressing tumors with a lower propensity to metastasize. Although clinical stage did not influence outcome in an early study, likely because of local disease progression, later findings showed significantly longer MST in dogs with stage I disease (637 days).^1^, ^3^ This finding is possibly related to differences in patient numbers, patient population and staging procedures.

Treatment also impacted survival in different ways. Surgery and the use of adjuvant chemotherapy were related to significantly longer survival, but the use of chemotherapy did not remain significant in the multivariate analysis. Although surgery acts on macroscopic disease and can palliate clinical signs, survival remained modest (in the range of 196 days), which is comparable to the MST of 137 days achieved in a previous study.^1^ Dogs treated with adjuvant chemotherapy compared to those receiving chemotherapy alone or no chemotherapy had a longer MST. In an early study, 5 dogs treated with some combination of surgery, radiation and adjuvant chemotherapy were reported to have a MST of 243 days.^10^ Previous studies also found a survival advantage for those dogs receiving a combination of surgery and chemotherapy (MST, 212 days) or chemotherapy and RT (MST, 355 days).^1^ The role of chemotherapy needs further investigation, but it might delay the onset of local or distant metastasis or both. Multiple antineoplastic agents were used, which included platinum compounds (carboplatin and cisplatin) in the majority of cases, TKIs (toceranib phosphate and imatinib mesylate), metronomic chemotherapy with cyclophosphamide, and anthracyclines (doxorubicin and mitoxantrone) as previously reported.^1^, ^3^ Carboplatin and cisplatin have been used to treat oral SCC based on reports in the human medical literature, and carboplatin also has been used in association with radiotherapy.^10^, ^17^, ^18^ Response to toceranib phosphate has been reported in solid tumors of dogs including head and neck carcinomas, with CR and PR reported in 12.5% and 62.5% of patients, respectively.^19^ In our cases, a few PR (n = 11) were recorded, although SD was most commonly reported (n = 16).

The use of RT was not significant, which could be a consequence of the low number of treated patients and the different protocols used (i.e., type II error). Therefore, its benefit remains unclear. A previous study also suggested that the addition of RT did not seem to influence prognosis. Similarly, in our study, RT was used palliatively and in association with antineoplastic agents in the majority of cases, and specifically as sole treatment in the gross disease setting in 2/20 cases and to treat residual disease in 8/20 cases.^3^ If radiation is available, it is likely to be beneficial in treating residual disease and may prolong survival if used in combination with chemotherapy or as an adjuvant to surgery.

The use of NSAIDs generally was associated with significantly longer survival. In a previous study, the majority of dogs received either NSAIDs alone or in combination with other treatments (e.g., surgery, chemotherapy or RT) and a survival benefit was seen.^1^ Cyclooxygenase (COX)‐2 expression has been shown in different types of carcinomas in dogs, including urothelial, prostatic, nasal, thyroid, mammary and anal sac carcinomas, among others.^20^, ^21^, ^22^, ^23^, ^24^, ^25^, ^26^, ^27^, ^28^, ^29^ Oral carcinomas in dogs also express COX‐2 receptors, but a specific study of tonsillar carcinomas has not yet been conducted.^30^ These tumors may express specific receptors or respond to anti‐inflammatory treatment because of the degree of inflammation associated with this tumor type, particularly in the gross disease setting. Previous measurable responses of non‐tonsillar SCC to piroxicam have been reported.^31^

In conclusion, our study suggests that asymptomatic patients, patients treated with surgery and adjuvant chemotherapy, and those receiving NSAIDs may have a better prognosis than untreated dogs or symptomatic patients, although overall survival remains short. Survival expectations for dogs with tonsillar carcinoma are limited, but the combination of multiple treatment modalities, whenever possible, is likely to be beneficial. Additional studies are needed to elucidate the role of surgery and different adjuvant treatments in the management of tonsillar carcinoma in dogs.

## CONFLICT OF INTEREST DECLARATION

Authors declare no conflict of interest.

## OFF‐LABEL ANTIMICROBIAL DECLARATION

Authors declare no off‐label use of antimicrobials.

## INSTITUTIONAL ANIMAL CARE AND USE COMMITTEE (IACUC) OR OTHER APPROVAL DECLARATION

Authors declare no IACUC or other approval was needed.

## HUMAN ETHICS APPROVAL DECLARATION

Authors declare human ethics approval was not needed for this study.
